# Annual Announcement of the Medical Department of the Pennsylvania College

**Published:** 1846-09

**Authors:** 


*Annual Announcement of the Medical Department of the Pennsylvania College. Session* 1846-7.

This institution has been in operation three years, and we are gratified to learn from the above Announcement, that it is in a most flourishing condition. Twenty-three students attended the lectures the first session; sixty the second, and ninety-four the third, showing a constant and very rapid increase. Philadelphia is certainly well supplied with medical schools, and all seem to be well attended.—*Balt. Ed.*

